# Supplementary material for: Comparison of the accuracy of diagnoses of oral potentially malignant disorders with dysplasia by a general dental clinician and a specialist using the Taiwanese Nationwide Oral Mucosal Screening Program
Source: PLoS One. 2021 Jan 11;16(1):e0244740. doi: 10.1371/journal.pone.0244740 (PMC7799778; doi:10.1371/journal.pone.0244740)
Supplement: S1 Data — (PDF) [file pone.0244740.s001.pdf]

Number	Sex	Age	Betel	Smk	Conscious	OPMDS	Diag_3	OPMDS_	OPMDS_
2	1	71	2	2	1.00	3	2	1.00	1.00
4	1	72	2	0	0.00	1	2	0.00	0.00
13	1	76	2	2	0.00	2	2	1.00	1.00
20	1	54	0	2	0.00	1	2	0.00	0.00
27	1	74	0	0	0.00	1	2	0.00	0.00
31	1	56	0	2	0.00	1	2	0.00	0.00
33	1	39	2	2	0.00	2	2	1.00	1.00
53	1	73	0	1	0.00	2	2	1.00	1.00
57	1	50	2	2	0.00	2	2	1.00	1.00
61	1	52	2	2	0.00	2	2	1.00	1.00
62	0	48	0	2	0.00	1	2	0.00	0.00
74	1	61	1	1	0.00	1	2	0.00	0.00
76	1	45	1	2	0.00		3	2.00	
80	1	67	0	1	0.00		3	2.00	
85	0	70	0	0	0.00	1	3	0.00	0.00
86	1	46	2	2	1.00		3	2.00	
88	1	48	2	2	0.00	1	2	0.00	0.00
74	1	61	1	1	0.00		2	2.00	
15	1	60	0	0	0.00		3	2.00	
17	0	82	0	0	0.00		3	2.00	
34	0	68	0	0	0.00	1	3	0.00	0.00
45	1	49	2	2	0.00		3	2.00	
60	1	44	2	2	0.00		3	2.00	
15	1	60	0	0	0.00		3	2.00	
17	0	82	0	0	0.00		3	2.00	
34	0	68	0	0	0.00	1	3	0.00	0.00
45	1	49	2	2	0.00		3	2.00	
60	1	44	2	2	0.00		3	2.00	
15	1	60	0	0	0.00		3	2.00	
17	0	82	0	0	0.00		3	2.00	
34	0	68	0	0	0.00	1	3	0.00	0.00
45	1	49	2	2	0.00		3	2.00	
60	1	44	2	2	0.00		3	2.00	
7	1	58	2	2	0.00		1	2.00	
14	1	40	2	2	1.00		1		
18	1	72	2	2	0.00	2	1	1.00	1.00
48	1	83	2	2	1.00	3	1	1.00	1.00
69	1	65	1	1	1.00		1		2.00
90	0	59	0	2	1.00		1		2.00
35	1	44	2	2	0.00	2	3	1.00	1.00
41	1	57	0	0	0.00	1	3	0.00	0.00
50	1	66	1	1	0.00	1	2	0.00	0.00
28	1	61	2	2	1.00		1		2.00
46	1	55	2	2	1.00	3	1	1.00	1.00
3	1	68	2	2	1.00	3	2	1.00	1.00
39	1	57	1	2	0.00	1	2	0.00	0.00
40	0	71	2	2	0.00	2	2	1.00	1.00
75	0	66	0	0	1.00	1	2	0.00	0.00
16	0	54	0	0	0.00	1	3	0.00	0.00
22	1	76	2	0	0.00	1	2	0.00	0.00
23	1	56	2	2	0.00	2	2	1.00	1.00

26	1	45	0	0	0.00	1	2	0.00	0.00
32	1	45	2	2	0.00	1	2	0.00	0.00
44	0	51	0	0	0.00	1	2	0.00	0.00
67	1	47	0	0	0.00	1	3	0.00	0.00
68	1	55	1	2	0.00	1	3	0.00	0.00
78	0	48	1	1	0.00	3	2	1.00	1.00
84	1	51	2	2	1.00		2	2.00	
94	1	53	2	2	0.00	2	2	1.00	1.00
64	1	47	1	1	0.00		3	2.00	
64	1	47	1	1	0.00		3	2.00	
64	1	47	1	1	0.00		3	2.00	
64	1	47	1	1	0.00		3	2.00	
72	1	79	1	1	1.00		3		2.00
6	1	70	0	0	1.00		1		2.00
12	1	56	0	0	1.00		2	2.00	
19	1	66	2	2	1.00	3	2	1.00	1.00
25	1	54	1	1	0.00	1	2	0.00	0.00
51	0	65	2	2	0.00	1	2	0.00	0.00
89	1	52	2	2	0.00	1	2	0.00	0.00
37	1	59	1	2	0.00	1	2	0.00	0.00
47	1	57	2	2	0.00	2	2	1.00	1.00
36	1	48	2	2	1.00	3	1	1.00	1.00
1	1	61	0	0	0.00	1	2	0.00	0.00
5	1	42	0	1	0.00	1	2	0.00	0.00
9	1	42	0	1	0.00	1	2	0.00	0.00
11	1	65	2	1	0.00	1	2	0.00	0.00
21	1	54	0	2	0.00	1	2	0.00	0.00
24	1	50	0	2	0.00	1	2	0.00	0.00
30	1	46	0	2	0.00	1	2	0.00	0.00
38	1	38	2	2	0.00	2	2	1.00	1.00
42	1	57	0	0	0.00		2	2.00	
43	1	54	2	2	0.00		2	2.00	
49	1	50	0	2	0.00	1	2	0.00	0.00
59	1	58	2	2	0.00	2	2	1.00	1.00
66	1	59	1	1	0.00	1	2	0.00	0.00
70	1	67	1	2	0.00	1	2	0.00	0.00
73	1	44	2	2	0.00	1	2	0.00	0.00
81	1	55	0	1	0.00		2	2.00	
82	1	53	2	2	0.00	1	2	0.00	0.00
83	1	52	1	1	0.00	1	2	0.00	0.00
91	0	65	0	0	0.00	1	2	0.00	0.00
92	1	68	1	2	0.00	1	2	0.00	0.00
93	1	74	0	1	0.00	1	2	0.00	0.00
81	1	55	0	1	0.00		2	2.00	
81	1	55	0	1	0.00		2	2.00	
81	1	55	0	1	0.00		2	2.00	
8	1	46	0	0	0.00		3	2.00	
29	1	64	2	1	0.00		3	2.00	
52	1	32	2	2	0.00		3	2.00	
58	1	81	2	2	0.00	1	3	0.00	0.00
63	1	59	1	2	0.00		3	2.00	
65	1	67	1	2	0.00		3	2.00	

77	1	55	1	2	0.00		3	2.00	
8	1	46	0	0	0.00		3	2.00	
29	1	64	2	1	0.00		3	2.00	
52	1	32	2	2	0.00		3	2.00	
58	1	81	2	2	0.00	1	3	0.00	0.00
63	1	59	1	2	0.00		3	2.00	
65	1	67	1	2	0.00		3	2.00	
77	1	55	1	2	0.00		3	2.00	
8	1	46	0	0	0.00		3	2.00	
58	1	81	2	2	0.00	1	3	0.00	0.00
65	1	67	1	2	0.00		3	2.00	
77	1	55	1	2	0.00		3	2.00	
65	1	67	1	2	0.00		3	2.00	
77	1	55	1	2	0.00		3	2.00	
29	1	64	2	1	0.00		3	2.00	
52	1	32	2	2	0.00		3	2.00	
63	1	59	1	2	0.00		3	2.00	
63	1	59	1	2	0.00		3	2.00	
54	1	59	0	2	0.00	1	3	0.00	0.00
71	1	54	2	2	0.00	1	3	0.00	0.00
10	1	57	0	1	0.00	1	2	0.00	0.00
55	1	28	0	2	0.00	1	2	0.00	0.00
56	0	60	1	2	0.00	2	2	1.00	1.00
79	1	61	1	1	1.00		3	2.00	
87	1	21	0	0	0.00		3	2.00	
79	1	61	1	1	1.00		3	2.00	
87	1	21	0	0	0.00		3	2.00	
79	1	61	1	1	1.00		3	2.00	
87	1	21	0	0	0.00		3	2.00	
79	1	61	1	1	1.00		3	2.00	
87	1	21	0	0	0.00		3	2.00	

OPMDS_2	Diag_OPM	Diag_OPM	Diag_COI	Diag_COI	Diag_COI	PRE_1	PGR_1	PRE_2	PGR_2
1.0	1.00	1.00		2.00	2.00				
0.0	1.00	1.00		2.00	2.00				
1.0	1.00	1.00		2.00	2.00				
0.0	1.00	1.00		2.00	2.00				
0.0	1.00	1.00		2.00	2.00				
0.0	1.00	1.00		2.00	2.00				
1.0	1.00	1.00		2.00	2.00				
1.0	1.00	1.00		2.00	2.00				
1.0	1.00	1.00		2.00	2.00				
1.0	1.00	1.00		2.00	2.00				
0.0	1.00	1.00		2.00	2.00				
0.0	1.00	1.00		2.00	2.00				
	1.00	1.00		2.00	2.00				
	1.00	1.00		2.00	2.00				
0.0	1.00	1.00		2.00	2.00				
	1.00	1.00		2.00	2.00				
0.0	1.00	1.00		2.00	2.00				
	1.00	1.00		2.00	2.00				
	2.00	2.00		2.00	2.00			0.00000	0.00
	2.00	2.00		2.00	2.00			0.00000	0.00
0.0	2.00	2.00		2.00	2.00			0.00000	0.00
	2.00	2.00		2.00	2.00			0.00000	0.00
	2.00	2.00		2.00	2.00			0.00000	0.00
	2.00	2.00		2.00	2.00			0.00000	0.00
	2.00	2.00		2.00	2.00			0.00000	0.00
0.0	2.00	2.00		2.00	2.00			0.00000	0.00
	2.00	2.00		2.00	2.00			0.00000	0.00
	2.00	2.00		2.00	2.00			0.00000	0.00
	2.00	2.00		2.00	2.00			0.00000	0.00
	2.00	2.00		2.00	2.00			0.00000	0.00
0.0	2.00	2.00		2.00	2.00			0.00000	0.00
	2.00	2.00		2.00	2.00			0.00000	0.00
	2.00	2.00		2.00	2.00			0.00000	0.00
		3.00		2.00	2.00			0.83333	1.00
		3.00		2.00	2.00			0.83333	1.00
1.0		3.00		2.00	2.00			0.83333	1.00
1.0		3.00		2.00	2.00			0.83333	1.00
		3.00		2.00				0.83333	1.00
		3.00		2.00				0.83333	1.00
1.0	1.00	1.00				1.00000	1.00		
0.0	1.00	1.00				1.00000	1.00		
0.0	1.00	1.00				1.00000	1.00		
		3.00				1.00000	1.00	0.83333	1.00
1.0		3.00				1.00000	1.00	0.83333	1.00
1.0	1.00	1.00	1.00	1.00	1.00				
0.0	1.00	1.00	1.00	1.00	1.00				
1.0	1.00	1.00	1.00	1.00	1.00				
0.0	1.00	1.00	1.00	1.00	1.00				
0.0	1.00	1.00	1.00	1.00	1.00				
0.0	1.00	1.00	1.00	1.00	1.00				
1.0	1.00	1.00	1.00	1.00	1.00				

0.0	1.00	1.00	1.00	1.00	1.00		
0.0	1.00	1.00	1.00	1.00	1.00		
0.0	1.00	1.00	1.00	1.00	1.00		
0.0	1.00	1.00	1.00	1.00	1.00		
0.0	1.00	1.00	1.00	1.00	1.00		
1.0	1.00	1.00	1.00	1.00	1.00		
	1.00	1.00	1.00	1.00	1.00		
1.0	1.00	1.00	1.00	1.00	1.00		
	2.00	2.00	1.00	1.00	1.00	0.00000	0.00
	2.00	2.00	1.00	1.00	1.00	0.00000	0.00
	2.00	2.00	1.00	1.00	1.00	0.00000	0.00
	2.00	2.00	1.00	1.00	1.00	0.00000	0.00
	1.00	1.00	1.00	1.00	1.00		
		3.00	1.00	1.00	1.00	0.83333	1.00
	1.00	1.00	1.00	1.00	1.00		
1.0	1.00	1.00	1.00	1.00	1.00		
0.0	1.00	1.00	1.00	1.00	1.00		
0.0	1.00	1.00	1.00	1.00	1.00		
0.0	1.00	1.00	1.00	1.00	1.00		
0.0	1.00	1.00	1.00	1.00	1.00		
1.0	1.00	1.00	1.00	1.00	1.00		
1.0		3.00	1.00	1.00	1.00	0.83333	1.00
0.0	1.00	1.00	1.00	1.00	1.00		
0.0	1.00	1.00	1.00	1.00	1.00		
0.0	1.00	1.00	1.00	1.00	1.00		
0.0	1.00	1.00	1.00	1.00	1.00		
0.0	1.00	1.00	1.00	1.00	1.00		
0.0	1.00	1.00	1.00	1.00	1.00		
0.0	1.00	1.00	1.00	1.00	1.00		
1.0	1.00	1.00	1.00	1.00	1.00		
	1.00	1.00	1.00	1.00	1.00		
	1.00	1.00	1.00	1.00	1.00		
0.0	1.00	1.00	1.00	1.00	1.00		
1.0	1.00	1.00	1.00	1.00	1.00		
0.0	1.00	1.00	1.00	1.00	1.00		
0.0	1.00	1.00	1.00	1.00	1.00		
0.0	1.00	1.00	1.00	1.00	1.00		
	1.00	1.00	1.00	1.00	1.00		
0.0	1.00	1.00	1.00	1.00	1.00		
0.0	1.00	1.00	1.00	1.00	1.00		
0.0	1.00	1.00	1.00	1.00	1.00		
0.0	1.00	1.00	1.00	1.00	1.00		
0.0	1.00	1.00	1.00	1.00	1.00		
	1.00	1.00	1.00	1.00	1.00		
	1.00	1.00	1.00	1.00	1.00		
	1.00	1.00	1.00	1.00	1.00		
	2.00	2.00	1.00	1.00	1.00	0.00000	0.00
	2.00	2.00	1.00	1.00	1.00	0.00000	0.00
	2.00	2.00	1.00	1.00	1.00	0.00000	0.00
0.0	2.00	2.00	1.00	1.00	1.00	0.00000	0.00
	2.00	2.00	1.00	1.00	1.00	0.00000	0.00
	2.00	2.00	1.00	1.00	1.00	0.00000	0.00

	2.00	2.00	1.00	1.00	1.00			0.00000	0.00
	2.00	2.00	1.00	1.00	1.00			0.00000	0.00
	2.00	2.00	1.00	1.00	1.00			0.00000	0.00
	2.00	2.00	1.00	1.00	1.00			0.00000	0.00
0.0	2.00	2.00	1.00	1.00	1.00			0.00000	0.00
	2.00	2.00	1.00	1.00	1.00			0.00000	0.00
	2.00	2.00	1.00	1.00	1.00			0.00000	0.00
	2.00	2.00	1.00	1.00	1.00			0.00000	0.00
	2.00	2.00	1.00	1.00	1.00			0.00000	0.00
0.0	2.00	2.00	1.00	1.00	1.00			0.00000	0.00
	2.00	2.00	1.00	1.00	1.00			0.00000	0.00
	2.00	2.00	1.00	1.00	1.00			0.00000	0.00
	2.00	2.00	1.00	1.00	1.00			0.00000	0.00
	2.00	2.00	1.00	1.00	1.00			0.00000	0.00
	2.00	2.00	1.00	1.00	1.00			0.00000	0.00
	2.00	2.00	1.00	1.00	1.00			0.00000	0.00
	2.00	2.00	1.00	1.00	1.00			0.00000	0.00
	2.00	2.00	1.00	1.00	1.00			0.00000	0.00
0.0	1.00	1.00	1.00	1.00	1.00				
0.0	1.00	1.00	1.00	1.00	1.00				
0.0	1.00	1.00	1.00	1.00	1.00				
0.0	1.00	1.00	1.00	1.00	1.00				
1.0	1.00	1.00	1.00	1.00	1.00				
	2.00	2.00	2.00	2.00	2.00	0.00000	0.00	0.00000	0.00
	2.00	2.00	2.00	2.00	2.00	0.00000	0.00	0.00000	0.00
	2.00	2.00	2.00	2.00	2.00	0.00000	0.00	0.00000	0.00
	2.00	2.00	2.00	2.00	2.00	0.00000	0.00	0.00000	0.00
	2.00	2.00	2.00	2.00	2.00	0.00000	0.00	0.00000	0.00
	2.00	2.00	2.00	2.00	2.00	0.00000	0.00	0.00000	0.00
	2.00	2.00	2.00	2.00	2.00	0.00000	0.00	0.00000	0.00
	2.00	2.00	2.00	2.00	2.00	0.00000	0.00	0.00000	0.00

Diag_COI	Diag_COI	Diag_COI	Pathology	Patho_allc	Diag	Diag_spec	Diag_COE_3type
12			2	1.00	3	1.00	2.00
12			2	1.00	5	1.00	2.00
12			2	1.00	3	1.00	2.00
12			2	1.00	3	1.00	2.00
12			2	1.00	6	1.00	2.00
12			2	1.00	6	1.00	2.00
12			2	1.00	2	1.00	2.00
12			2	1.00	2	1.00	2.00
12			2	1.00	3	1.00	2.00
12			2	1.00	3	1.00	2.00
12			2	1.00	6	1.00	2.00
12			2	1.00	6	1.00	2.00
12			3	2.00	7	1.00	2.00
12			3	2.00	7	1.00	2.00
10			2	1.00	9	1.00	2.00
12			3	2.00	7	1.00	2.00
12			2	1.00	4	1.00	2.00
12			3	2.00	6	1.00	2.00
10			3	2.00	10	2.00	2.00
10			3	2.00	10	2.00	2.00
10			2	1.00	10	2.00	2.00
10			3	2.00	10	2.00	2.00
10			3	2.00	10	2.00	2.00
10			3	2.00	10	2.00	2.00
10			3	2.00	10	2.00	2.00
10			2	1.00	10	2.00	2.00
10			3	2.00	10	2.00	2.00
10			3	2.00	10	2.00	2.00
10			3	2.00	10	2.00	2.00
10			3	2.00	10	2.00	2.00
10			2	1.00	10	2.00	2.00
10			3	2.00	10	2.00	2.00
10			3	2.00	10	2.00	2.00
12			3	2.00	1		2.00
12			1		1		2.00
12			2	1.00	1		2.00
10			2	1.00	1		2.00
2			1		1		2.00
2			1		1		2.00
1	3.00		2	1.00	7	1.00	1.00
1	3.00		2	1.00	7	1.00	1.00
1	3.00		2	1.00	4	1.00	1.00
1	3.00		1		1		1.00
1	3.00		2	1.00	1		1.00
3	1.00	1.00	2	1.00	2	1.00	2.00
3	1.00	1.00	2	1.00	2	1.00	2.00
3	1.00	1.00	2	1.00	3	1.00	2.00
3	1.00	1.00	2	1.00	2	1.00	2.00
4	1.00	1.00	2	1.00	9	1.00	2.00
4	1.00	1.00	2	1.00	3	1.00	2.00
4	1.00	1.00	2	1.00	3	1.00	2.00

4	1.00	1.00	2	1.00	3	1.00	2.00
4	1.00	1.00	2	1.00	5	1.00	2.00
4	1.00	1.00	2	1.00	3	1.00	2.00
4	1.00	1.00	2	1.00	9	1.00	2.00
4	1.00	1.00	2	1.00	8	1.00	2.00
4	1.00	1.00	2	1.00	3	1.00	2.00
4	1.00	1.00	3	2.00	3	1.00	2.00
4	1.00	1.00	2	1.00	3	1.00	2.00
4	1.00	1.00	3	2.00	10	2.00	2.00
4	1.00	1.00	3	2.00	10	2.00	2.00
4	1.00	1.00	3	2.00	10	2.00	2.00
4	1.00	1.00	3	2.00	10	2.00	2.00
5	1.00	1.00	1		7	1.00	2.00
5	1.00	1.00	1		1		2.00
6	1.00	1.00	3	2.00	5	1.00	2.00
6	1.00	1.00	2	1.00	3	1.00	2.00
6	1.00	1.00	2	1.00	4	1.00	2.00
6	1.00	1.00	2	1.00	4	1.00	2.00
6	1.00	1.00	2	1.00	4	1.00	2.00
7	1.00	1.00	2	1.00	5	1.00	2.00
7	1.00	1.00	2	1.00	5	1.00	2.00
7	1.00	1.00	2	1.00	1		2.00
8	1.00	1.00	2	1.00	6	1.00	2.00
8	1.00	1.00	2	1.00	6	1.00	2.00
8	1.00	1.00	2	1.00	6	1.00	2.00
8	1.00	1.00	2	1.00	3	1.00	2.00
8	1.00	1.00	2	1.00	3	1.00	2.00
8	1.00	1.00	2	1.00	3	1.00	2.00
8	1.00	1.00	2	1.00	4	1.00	2.00
8	1.00	1.00	2	1.00	3	1.00	2.00
8	1.00	1.00	3	2.00	6	1.00	2.00
8	1.00	1.00	3	2.00	6	1.00	2.00
8	1.00	1.00	2	1.00	6	1.00	2.00
8	1.00	1.00	2	1.00	3	1.00	2.00
8	1.00	1.00	2	1.00	6	1.00	2.00
8	1.00	1.00	2	1.00	6	1.00	2.00
8	1.00	1.00	2	1.00	6	1.00	2.00
8	1.00	1.00	3	2.00	6	1.00	2.00
8	1.00	1.00	2	1.00	6	1.00	2.00
8	1.00	1.00	2	1.00	4	1.00	2.00
8	1.00	1.00	2	1.00	6	1.00	2.00
8	1.00	1.00	2	1.00	6	1.00	2.00
8	1.00	1.00	2	1.00	3	1.00	2.00
8	1.00	1.00	3	2.00	6	1.00	2.00
8	1.00	1.00	3	2.00	6	1.00	2.00
8	1.00	1.00	3	2.00	6	1.00	2.00
8	1.00	1.00	3	2.00	10	2.00	2.00
8	1.00	1.00	3	2.00	10	2.00	2.00
8	1.00	1.00	3	2.00	10	2.00	2.00
8	1.00	1.00	2	1.00	10	2.00	2.00
8	1.00	1.00	3	2.00	10	2.00	2.00
8	1.00	1.00	3	2.00	10	2.00	2.00

8	1.00	1.00	3	2.00	10	2.00	2.00
8	1.00	1.00	3	2.00	10	2.00	2.00
8	1.00	1.00	3	2.00	10	2.00	2.00
8	1.00	1.00	3	2.00	10	2.00	2.00
8	1.00	1.00	2	1.00	10	2.00	2.00
8	1.00	1.00	3	2.00	10	2.00	2.00
8	1.00	1.00	3	2.00	10	2.00	2.00
8	1.00	1.00	3	2.00	10	2.00	2.00
8	1.00	1.00	3	2.00	10	2.00	2.00
8	1.00	1.00	2	1.00	10	2.00	2.00
8	1.00	1.00	3	2.00	10	2.00	2.00
8	1.00	1.00	3	2.00	10	2.00	2.00
8	1.00	1.00	3	2.00	10	2.00	2.00
8	1.00	1.00	3	2.00	10	2.00	2.00
8	1.00	1.00	3	2.00	10	2.00	2.00
8	1.00	1.00	3	2.00	10	2.00	2.00
8	1.00	1.00	3	2.00	10	2.00	2.00
8	1.00	1.00	3	2.00	10	2.00	2.00
9	1.00	1.00	2	1.00	8	1.00	2.00
9	1.00	1.00	2	1.00	8	1.00	2.00
11	1.00	1.00	2	1.00	3	1.00	2.00
11	1.00	1.00	2	1.00	3	1.00	2.00
11	1.00	1.00	2	1.00	3	1.00	2.00
13	2.00	2.00	3	2.00	10	2.00	3.00
13	2.00	2.00	3	2.00	10	2.00	3.00
13	2.00	2.00	3	2.00	10	2.00	3.00
13	2.00	2.00	3	2.00	10	2.00	3.00
13	2.00	2.00	3	2.00	10	2.00	3.00
13	2.00	2.00	3	2.00	10	2.00	3.00
13	2.00	2.00	3	2.00	10	2.00	3.00
13	2.00	2.00	3	2.00	10	2.00	3.00

Variable	Label	Value label
Pathology report	same as left(Gold standard)	1 Cancer
		2 Dysplasia
		3 Others
OPMDs	Oral potentially malignant disor	1 mild
		2 moderate
		3 servere
Diag_primary	diagnosis of primary examiner	1 Suspected oral cancer
		2 Unexplained persistent mass
		3 Erythroplakia
		4 Erythroleukoplakia
		5 Verrucous hyperplasia
		6 Non-homogeneous leukoplakia
		7 Thick homogeneous leukoplakia
		8 Thin homogeneous leukoplakia
		9 Submucous fibrosis
		10 Unhealed ulceration for > 2 weeks
		11 Lichen planus
		12 Abnormal mucosa without diagnosis
		13 Other
Diag_specialist	diagnosis of specialist examiner	1 Suspected oral cancer
		2 Unexplained persistent mass
		3 Erythroplakia
		4 Erythroleukoplakia
		5 Verrucous hyperplasia
		6 Non-homogeneous leukoplakia
		7 Thick homogeneous leukoplakia
		8 Thin homogeneous leukoplakia
		9 Submucous fibrosis
		10 Unhealed ulceration for > 2 weeks
		11 Lichen planus
		12 Abnormal mucosa without diagnosis
		13 Other
Diag_COE_new	diagnosis of primary examiner i	1 OPMD
		2 other
		3 cancer
Diag_COE_alldysorother	diagnosis of primary examiner i	1 OPMD
		2 other
Diag_special_alldysorother	diagnosis of specialist examiner	1 OPMD
		2 other
Patho_alldysorother	pathology report	1 dysplasia
		2 other
